# Accuracy of McMonnies Questionnaire as a Screening Tool for Chinese Ophthalmic Outpatients

**DOI:** 10.1371/journal.pone.0153047

**Published:** 2016-04-13

**Authors:** Furong Tang, Jiwei Wang, Zheng Tang, Mei Kang, Qinglong Deng, Jinming Yu

**Affiliations:** 1 Institute of Epidemiology and Health Statistics, School of Public Health, Fudan University, Shanghai, China; 2 Collaborative Innovation Center of Social Risks Governance in Health, Fudan University, Shanghai, China; University of Florence, ITALY

## Abstract

**Objective:**

To evaluate the accuracy of the McMonnies questionnaire (MQ) as a screening tool for dry eye (DE) among Chinese ophthalmic outpatients.

**Methods:**

We recruited 27718 cases from 94 hospitals (research centers), randomly selected from 45 cities in 23 provinces from July to November in 2013. Only symptomatic outpatients were included and they were in a high risk of DE. Outpatients meeting the criteria filled out questionnaires and then underwent clinical examinations by qualified medical practitioners. We mainly evaluated sensitivity, specificity, diagnostic odds ratio (DOR), and area under the receiver-operating characteristic curve (AUC) to evaluate the accuracy of the questionnaire in the diagnosis of dry eye.

**Results:**

Of all the subjects included in the study, sensitivity, specificity, and DOR were 0.77, 0.86 and 20.6, respectively. AUC was 0.865 with a 95% CI (0.861, 0.869). The prevalence of DE among the outpatients claiming “constantly” as the frequency of symptom was over 90%. Scratchiness was a more accurate diagnostic indication than dryness, soreness, grittiness or burning. Different cut points of McMonnies Index (MI) scores can be utilized to optimize the screening results.

**Conclusions:**

MQ can be an effective screening tool for dry eye. We can take full advantage of MI score during the screening process.

## Introduction

The most accepted definition of Dry Eye Disease (DED) was provided by the International Dry Eye Workshop in 2007, referring to it as a multifactorial disease of the tears and ocular surface that results in symptoms of discomfort, visual disturbance, and tear film instability, with potential damage to the ocular surface. It is accompanied by increased osmolarity of the tear film and inflammation of the ocular surface [1]. This definition includes six sequelae: visual compromise, symptoms of discomfort, ocular surface damage, tear film instability, inflammation and increased osmolarity, making dry eye a disease diagnosed by both clinical symptoms and signs.

The prevalence of dry eye based on large epidemiological studies varies from 5.5% to 33.7%, and Asians are more susceptible than Caucasians [2–8]. However, different diagnosis criteria have been applied in these studies. At present, the diagnosis of dry eye is based on clinical tests and questionnaires, but thus far there is no “gold standard”. No single clinical test can be used as a standard criterion for diagnosis, nor has a combination of clinical tests been universally accepted to differentiate DE from healthy eyes.

In spite of the subjective nature of self-reported symptoms, they are more reliable and repeatable than objective clinical tests in detecting dry eye [9]. MQ has been found to be a useful screening instrument, providing valid sensitivity and specificity information [10]. This questionnaire is composed of 14 questions focusing on the risk factors for DED. Categories of assessment include demographic information (gender and age), dry eye symptoms, previous and current dry eye treatments, secondary symptoms (associated with environmental stimuli), systemic conditions (Sjögren syndrome, arthritis, thyroid disease), and dryness of the mucous membranes (chest, throat, mouth or vagina) [11].

Although some studies [12–16] have reported correlations among symptoms and clinical tests, and some researchers [16, 17] have validated the questionnaire in some populations, the formal assessment of MQ as a screening instrument for detecting DE in Chinese ophthalmic outpatients is unprecedented.

What’s more, few studies pay attention to utilizing the MI scores to optimize the screening results. As outpatients in this study are in high risk of DE, we lower the cut-offs of MI scores to maximize sensitivity and compare the accuracy under different circumstances. We can also observe the distributions of DE and non-DE groups according MI scores.

## Materials and Methods

### Outpatient recruitment

Ninety-four hospitals (research centers) were randomly selected from 45 cities in 23 provinces from July to November in 2013. From these hospitals (research centers), we recruited 27718 outpatients from ophthalmic clinics by registration orders. Inclusion criterion was a presence of at least one of the six symptoms: dry sensation, foreign body sensation, burning sensation, eyesight fatigue, discomfort, and vision fluctuation. Outpatients with other eye diseases such as conjunctivitis, glaucoma, and ocular trauma were excluded. The rest filled in the MQ and underwent clinical examinations including Tear breakup time tests, Schirmer I tests and Fluorescein staining by trained medical practitioners. This survey was approved by the Institutional Review Board (IRB) of Fudan University. The investigation was conducted in strict accordance with the principles expressed in the Declaration of Helsinki. Details and procedures of this study were indicated to all the patients by practitioners before the questionnaire and clinical tests. Oral consents were sought from all subjects in advance. Participant would be excluded in the absence of agreement, thus in this way we documented and insured all patient consents. Both the collection and analysis of the data were anonymous, which explained why we used oral consents instead of written consents. In addition, the clinical tests did not cause any physical harm to patients. We believed nothing was against health, safety and privacy of patients in this survey.

### McMonnies Index

The full version of the McMonnies questionnaire is available in S1 Appendix, with the full set of weighting scores for each question. Scores are tabulated using a weighted-point assignment “based on clinical experience”, where all scores are summed, with weights obtained to calculate an overall “Index” [18]. The Index ranges from 0 to 45, where a higher score is regarded as more indicative of DED [19]. A cut-point of greater than 14.5 is recommended for a dry eye diagnosis [19].

### Diagnosis of dry eye disease

Diagnosis was established according to a consensus of Chinese dry-eye diagnostic criteria from the Chinese Medical Association as follows: (1) presence of at least one of the six symptoms: dry sensation, foreign body sensation, burning sensation, eyesight fatigue, discomfort and vision fluctuation; (2) TBUT≤5s or Schirmer I test (without anesthesia) ≤5mm/5min; (3) a positive diagnosis of fluorescein staining accompanied by one of the results: 5s<TBUT≤10s or 5mm/5min< Schirmer I test (without anesthesia) ≤10mm/5min. The presence of (1) was essential for disease diagnosis. Subjects showing the presence of a combination of (1) and (2), or (1) and (3) were diagnosed with DED.

### Statistics analysis

Data analysis was performed using the SPSS 19.0 software. Student’s t-tests and ANOVA tests were utilized for quantitative variables. The Chi-squared test was utilized for qualitative variables. Trend tests were conducted to verify if there were ascending or descending trends in quantitative variables. Values of p≤0.05 were considered to be statistically significant. Main values used to assess the accuracy in detecting DED included sensitivity, specificity, DOR, and AUC. The 95% confidence intervals for the AUC were also evaluated.

## Results

Overall, the sensitivity, specificity, false negative rate, and false positive rate of MQ were 0.77, 0.86, 0.23, and 0.14, respectively. The positive likelihood ratio, the negative likelihood ratio and DOR were 5.47, 0.27, and 20.6, respectively. AUC was 0.865. The 95% CI of AUC was (0.861, 0.869). A fourfold table of diagnosis results across the study population can be found in S1 Text.

Demographic data, average MI scores, rates of MI>14.5 (i.e positive diagnosis by MQ) and positive rates as diagnosed by the gold standard we adopted in this study are summarized in Table 1. A significantly higher average score was observed among females than males, 16.3 versus 13.7(p<0.01). A rising trend (p<0.01) in average scores with age was observed. The highest average scores and positive diagnostic rates using both methods (p<0.05) were observed among outpatients who reported dryness. The highest average scores and positive diagnostic rates using both methods (p<0.05) were observed among outpatients who reported more than three symptoms. The prevalence of the outpatients reporting “constantly” as the frequency of symptom was 94.5%.

**Table 1:** Demographic data, average scores of MI and positive diagnostic rate by MQ and clinical test.

	N(%)	Average MIScore^1^	p	MI>14.5		Diagnosed by clinical tests	
		(mean ± sd)		No.	positive rate(95%CI)	No.	positive rate (95%CI)
**Gender**							
**Male**	13525(48.7)	13.7±5.9		6650	0.492(0.483,0.500)	8497	0.628(0.620,0.636)
**Female**	14256(51.3)	16.3±6.3	P_(t tes)t_<0.01	6875	0.635(0.627,0.643)	10362	0.727(0.720,0.734)
**Age**							
**<25**	5966(21.5)	11.7±6.0		2267	0.380(0.368,0.392)	3240	0.543(0.530,0.556)
**25–45**	12469(44.9)	14.7±5.6		6627	0.532(0.523,0.540)	8222	0.659(0.651,0.668)
**>45**	9346(33.6)	17.6±6.0	P_(trend)_<0.01	6804	0.728(0.719,0.737)	7397	0.792(0.783,0.800)
**Symptom**^2^							
**Soreness**	430(1.55)	13.8±6.4		191	0.444(0.397,0.491)	214	0.498(0.450,0.545)
**Scratchiness**	1299(4.68)	12.8±6.7		503	0.387(0.361,0.414)	672	0.517(0.490,0.545)
**Dryness**	7862(28.3)	15.0±5.9		4316	0.549(0.538,0.560)	5248	0.668(0.657,0.678)
**Grittiness**	1645(5.92)	14.0±6.6		736	0.447(0.423,0.472)	958	0.582(0.559,0.606)
**Burning**	1185(4.27)	13.6±6.6	P_(anova)_<0.01	463	0.391(0.363,0.419)	676	0.571(0.542,0.599)
**No. of symptoms**							
**0**	2040(7.34)	12.1±6.4		896	0.439(0.418,0.461)	1229	0.602(0.581,0.624)
**1**	12421(44.7)	14.5±6.2		6209	0.500(0.491,0.509)	7768	0.625(0.617,0.634)
**2**	6899(24.8)	15.2±5.9		3918	0.568(0.556,0.580)	4720	0.684(0.673,0.695)
**3–5**	6421(23.1)	17.1±5.6	P_(trend)_<0.01	6899	0.728(0.716,0.739)	5142	0.801(0.791,0.811)
**Frequency of symptom**							
**Never**	2003(7.21)	9.9±5.8		433	0.216(0.198,0.234)	844	0.421(0.400,0.443)
**Sometimes**	12204(43.9)	12.5±5.5		4416	0.362(0.353,0.370)	6654	0.545(0.536,0.554)
**Often**	11821(42.6)	17.8±5.0		9192	0.778(0.770,0.785)	9693	0.820(0.813,0.827)
**Constantly**	1753(6.31)	20.9±5.3	P_(trend)_<0.01	1657	0.945(0.935,0.956)	1668	0.952(0.941,0.962)
**Total**	27781(100)	15.1±6.2		15698	0.565(0.559,0.571)	18859	0.679(0.673,0.684)

^1^MI: McMonnies Index

^2^Symptom: outpatients reporting one single symptom

Table 2 is a detailed evaluation of the MQ among different subgroups. A rising trend in sensitivity was observed with age among the <25y age group, 25-45y age group and >45y age group, while the reverse was true regarding specificity, DOR and AUC.

**Table 2:** Evaluation of accuracy of MQ for different subgroups.

	sensitivity	specificity	DOR^1^	AUC^2^	95% CI^3^of AUC	
					lower	upper
Gender						
male	0.717	0.890	20.5	0.804	0.796	0.811
female	0.806	0.821	19.1	0.814	0.805	0.822
Age						
<25	0.645	0.935	26.1	0.790	0.778	0.802
25–45	0.741	0.874	19.8	0.807	0.799	0.815
>45	0.847	0.725	14.6	0.786	0.774	0.799
Symptom^4^						
Soreness	0.710	0.891	20.0	0.807	0.766	0.849
Scratchiness	0.689	0.936	32.4	0.855	0.834	0.875
Dryness	0.750	0.854	17.5	0.858	0.850	0.866
Grittiness	0.686	0.885	16.8	0.835	0.816	0.855
Burning	0.627	0.923	20.1	0.822	0.798	0.845
No. of symptoms						
0	0.697	0.963	59.9	0.896	0.883	0.910
1	0.725	0.876	18.6	0.852	0.845	0.859
2	0.738	0.799	11.2	0.834	0.824	0.844
3–5	0.864	0.817	28.4	0.894	0.884	0.904
Frequency of symptom						
Never	0.435	0.943	0.378	0.689	0.664	0.713
Sometimes	0.600	0.924	0.524	0.762	0.753	0.771
Often	0.877	0.674	0.551	0.776	0.763	0.788
constantly	0.953	0.200	0.153	0.576	0.508	0.645

^1^DOR: diagnostic odds ratio

^2^AUC: area under the receiver-operating characteristic curve

^3^95% CI: 95% confidence intervals

^4^Symptom reported: outpatients reporting one single symptom

Addressing symptom reported, a much higher DOR (32.4) was observed for the group reporting scratchiness than any other group. The AUC of scratchiness (0.855) group was smaller than dryness group (0.858), but larger than grittiness (0.835), soreness (0.807), or burning group (0.822).

As the number of symptoms increased, sensitivity increased, while specificity trended to fall basically. The greatest DOR (59.9) and largest AUC (0.896) were observed for the group reporting 0 symptom. But we did not find a trend of DOR or AUC when the number of symptoms increased.

As the frequency of symptoms increased, sensitivity increased, while specificity had an opposite trend. The greatest DOR was observed in the group reporting symptoms “sometimes” (18.2), followed by “often” (14.7), “never” (12.7) and “constantly” (5.07). The highest AUC was found in the “often” group (0.843), followed by “sometimes” (0.822), “never” (0.764) and “constantly” (0.708).

Table 3 shows sensitivity, specificity and DOR at different cut-offs for different subgroups. Basically a rising trend of sensitivity was observed as the MI cut-offs went high in each classification, while the specificity and DOR trended to fall. But the trend of DOR was uncertain among the subgroups of frequency of symptom.

**Table 3:** Sensitivity, specificity and DOR at different MI score cut-offs.

	sensitivity			specificity			DOR^1^		
	MI^2^>6.5	MI>10.5	MI>14.5	MI>6.5	MI>10.5	MI>14.5	MI>6.5	MI>10.5	MI>14.5
Gender									
male	0.959	0.869	0.717	0.260	0.600	0.890	8.21	9.95	20.5
female	0.984	0.926	0.806	0.183	0.491	0.821	13.8	12.1	19.1
Age									
<25	0.933	0.811	0.645	0.401	0.738	0.935	9.32	12.1	26.1
25–45	0.973	0.894	0.741	0.168	0.496	0.874	7.28	8.30	19.8
>45	0.990	0.946	0.847	0.108	0.416	0.725	12.0	12.5	14.6
Symptom reported^3^									
Soreness	0.967	0.855	0.710	0.199	0.523	0.891	7.28	6.47	20.0
Scratchiness	0.948	0.830	0.689	0.325	0.619	0.936	8.78	7.93	32.4
Dryness	0.980	0.900	0.750	0.194	0.481	0.854	11.8	8.34	17.5
Grittiness	0.965	0.860	0.686	0.230	0.575	0.885	8.24	8.31	16.8
Burning	0.951	0.815	0.627	0.191	0.639	0.923	4.58	7.80	20.1
No. of symptoms									
0	0.930	0.815	0.697	0.518	0.827	0.963	14.3	21.1	59.9
1	0.972	0.881	0.725	0.217	0.530	0.876	9.62	8.35	18.6
2	0.969	0.896	0.738	0.171	0.531	0.799	6.45	9.75	11.2
3–5	0.988	0.954	0.864	0.170	0.497	0.817	16.9	20.5	28.4
Frequency of symptom									
Never	0.858	0.636	0.435	0.443	0.748	0.943	4.81	5.19	12.7
Sometimes	0.944	0.808	0.600	0.250	0.614	0.924	5.62	6.69	18.2
Often	0.998	0.970	0.877	0.056	0.305	0.674	29.6	14.2	14.7
Constantly	1.000	0.995	0.953	0	0.071	0.200	1.94^4^	15.2	5.07

^1^DOR: diagnostic odds ratio

^2^MI: McMonnies Index

^3^Symptom reported: outpatients reporting one single symptom

^4^1.94: If a fourth fold table contains 0, the DOR will be undefined. Under this circumstance the method to get an approximation of DOR is to add 0.5 to all counts in the table [20, 21].

**Fig 1:**
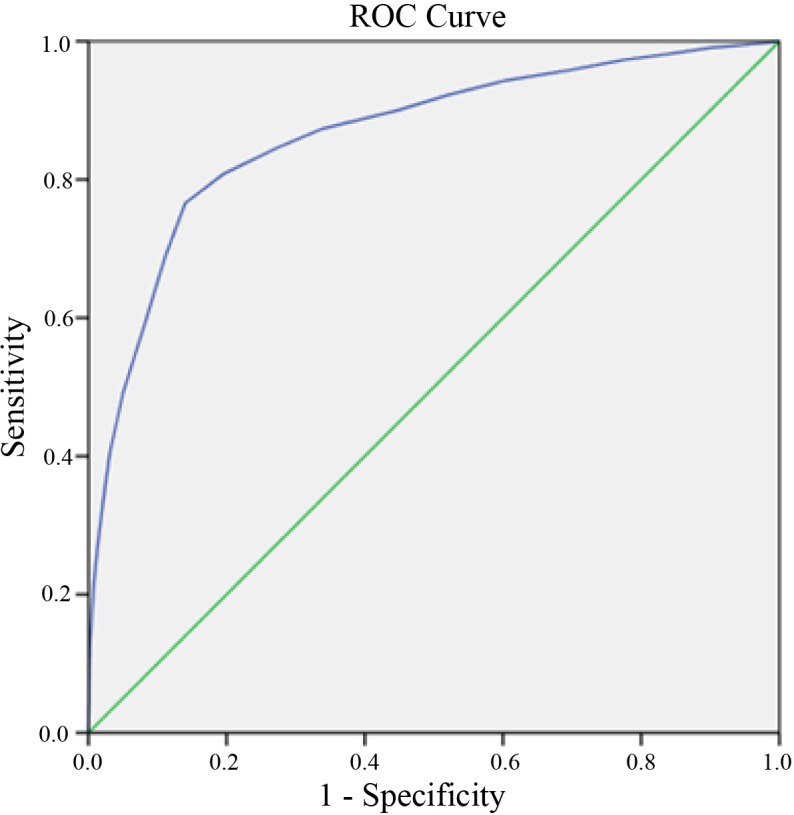
The receiver operating characteristic curve.

ROC is plotted in Fig 1. Peak Youden’s index (0.625) was found when MI was 14.5, with sensitivity of 0.766 and specificity of 0.860 in Table 4. AUC was 0.865 with a 95% CI from 0.861 to 0.869.

**Table 4:** Sensitivity, specificity and Youden’s Index for different MI scores.

MI^1^	sensitivity	specificity	Youden’s Index
7.5	0.973	0.226	0.199
8.5	0.958	0.306	0.264
9.5	0.943	0.396	0.339
10.5	0.922	0.479	0.401
11.5	0.900	0.552	0.452
12.5	0.873	0.663	0.536
13.5	0.845	0.728	0.573
14.5	0.808	0.804	0.612
15.5	0.766	0.860	0.626
16.5	0.687	0.890	0.577
17.5	0.591	0.919	0.510
18.5	0.491	0.950	0.441
19.5	0.406	0.969	0.375
20.5	0.334	0.979	0.313
21.5	0.266	0.987	0.253

^1^MI: McMonnies Index

The main object of this table was to find out the peak Youden’s Index. The index increased when MI got closer to 14.5. Therefore, only necessary data was showed here. The complete table can be found in S2 Text.

**Fig 2:**
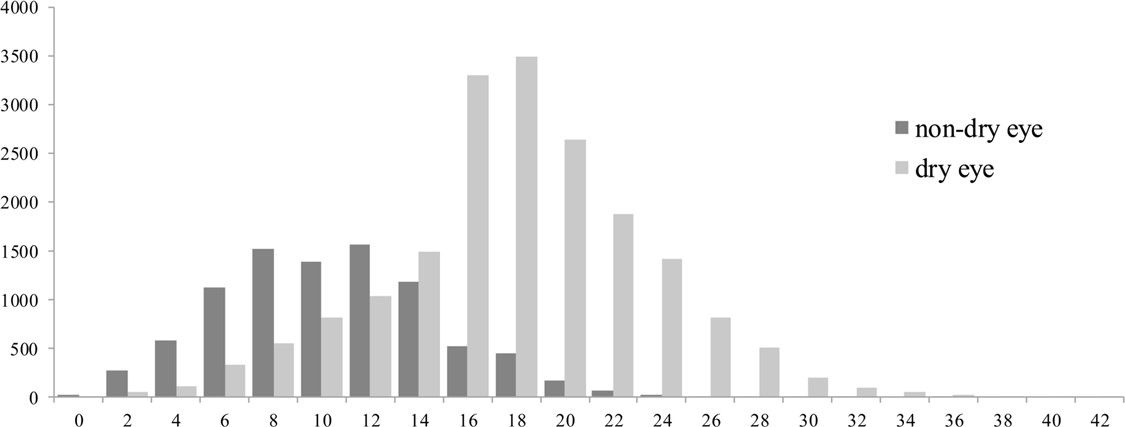
Distributions of DE and non-DE groups according to MI. X axes: McMonnies Index. Y axes: the number of subjects.

An obvious overlap between the DE and non-DE groups was found between MI scores of 10 to 14 in Fig 2. The distribution of DE subjects concentrated in the range from 14 to 24, while non-DE subjects mainly concentrated in the range from 6 to 14.

## Discussion

Symptom assessment is a critical component for the diagnosis for dry eye [22–24] and can be a very effective screening tool. Supporting the potential utility of this approach, a study reported that screening based on symptoms alone could better discriminate DE from non-DE than one combining symptoms and diagnosed sign [25]. For screening or research based on large populations, methods for diagnosing dry eye must be economically viable, noninvasive and brief, making questionnaires a favorable option. Many questionnaires have been used in epidemiological studies, serving as screening tools [3, 4, 6, 8, 26–28] or to grade the severity of dry eye [29–31].

In summary, this study indicated good accuracy of the questionnaire in distinguishing DE and non-DE, supported by the sensitivity, specificity, DOR and AUC results. The spectrum of sensitivity and specificity has varied in different studies. Discriminant analyses of one investigation by McMonnies [10] reported a 98% sensitivity and a 97% specificity. These results were proven to be biased estimates because they stemmed from the same data from which the classification process was developed [32]. Later, in another research by McMonnies [19], sensitivity and specificity were found to be 92% and 93%, respectively. The study by Kelly K. Nichols [18] yielded a sensitivity of 82% and specificity of 36%, indicating a comparatively low specificity. Another large scale epidemiological study [27] focusing on US women reported a sensitivity of 77% and specificity of 86%, which were quite close to the results of our study. There are several possible reasons for the divergence of our results from some other existing investigations. Firstly, our inclusion criterion in this study was ophthalmic outpatients with at least one of the six symptoms, inferring that these subjects may be at high risk for dry eye. Secondly, the prevalence of dry eye is different among races, and we focused on a different race from previous studies. Thirdly, throughout our study, a strict gold standard was employed for prudent diagnosis results.

Peak Youden’s Index was found at the cut-point of 14.5 MI, in accordance with another study focusing on the white race [19], suggesting that this cut-point is also suitable for Chinese ophthalmic outpatients. Although the cut-point of 14.5 is used as a diagnostic criterion in general practice, the results of MI scores may carry more diagnostic information and could offer potential advantages. For instance, in a study [16] MI was used to divide people into normal (MI<10), moderate dry eye (10≤MI≤20) and severe dry eye (MI>20) groups, implying that MI could reflect disease severity to some degree. On the other hand, a negative result for a screening test that has a very high sensitivity can rule the patients out (SnOUT) [33]. Therefore, in order to increase the referral rates of DE patients to clinical assessment, we can lower MI thresholds to maximize sensitivity. In Table 3, sensitivity went extremely high when we lowered the MI cut-off to 6.5, inferring that we would miss few DE patients under this situation. The sensitivity even reached 100% for the group claiming the frequency of symptom as “constantly” at the cut-off of 6.5 in this sample.

Fig 2 hints that MQ shows dissatisfactory diagnostic capacity when MI scores range from 10 to 14. We tentatively put forward that the accuracy of the MQ reduces when MI gets closer to the cut-point of 14.5. In another investigation by McMonnies [19], 10≤MI≤20 was defined as a equivocal classification group, due to an overlap of DE and non-DE subjects, which is different from Fig 2. The discrepancy is probably a consequence of the proportional difference in the DE and non-DE groups in the two studies. It has even been suggested by McMonnies [19] that those subjects with MI scores between 10 and 20 should be removed from the study when involving MQ diagnosis results. However, in this survey, all eligible subjects were included because the object of this study was to assess the accuracy of MQ under actual outpatient situations.

The accuracy of the questionnaire becomes less reliable with aging, supported by DORs and AUCs in Table 4. We also find the greatest DOR for the group reporting scratchiness, implying that this symptom may be more reliable than the other four symptoms in detecting DE (Table 4). The MQ performs best in the group reporting no symptom, with a greater DOR (59.9) than outpatients with symptom(s).

Based on all the discussion above, we put forward some suggestions on the usage of the MQ in screening for DE. Firstly, to avoid missing DE patients, we can lower the MI score threshold when necessary. Secondly, prevalence of DE among different subgroups will assist us during the process of screening. For instance, as the prevalence of outpatients claiming “constantly” as the frequency of symptoms is over 90% in this study, we suggest referrals for all these outpatients to clinical assessment. Thirdly, special attention should be paid to the subgroups proven to be more accurate in diagnose.

We have to admit that this survey has some limitations. Firstly, this study was not designed to test the reliability and validity. Thus we did not recruit the outpatients on two occasions and could not assess test-retest reliability or validity. Secondly, disease severity was not taken into consideration. Finally, the extension of our conclusions is restricted by differing gold standards applied in different studies, which is a universal problem among dry eye surveys.

The major strength of this survey was its large sample size. Compared to other parallel studies [18, 34] recruiting less than 300 subjects each, this epidemiological study recruited 27781 outpatients. Secondly, the representation of the sample was assured, because the subjects were from different provinces nationwide. Finally, the assessment of the MQ in large Chinese outpatient samples has not yet been reported, filling an important blank space in the relevant research area.

To conclude, the MQ is an effective screening tool for DED in Chinese outpatients. Based on the results obtained from this epidemiological study, ophthalmologists can employ the questionnaire during the process of preliminary diagnosis. The detailed results will further assist them in determining more valuable and accurate diagnostic information. In addition, epidemiologists can apply it in large population screening, dramatically reducing the cost. Further studies of the assessment of MQ are warranted to evaluate the relationship between the disease severity and the MI.

## Supporting Information

S1 Appendix The full version of the McMonnies questionnaire. (PDF)

S1 Data Minimal data set used to reach the conclusions drawn in the manuscript with related metadata and methods. (XLSX)

S1 Text A fourfold table of diagnosis results across the study population. (PDF)

S2 Text The complete table for sensitivity, specificity and Youden’s Index for different MI scores. (PDF)
