# Supplementary material for: Accuracy of McMonnies Questionnaire as a Screening Tool for Chinese Ophthalmic Outpatients
Source: PLoS One. 2016 Apr 13;11(4):e0153047. doi: 10.1371/journal.pone.0153047 (PMC4830624; doi:10.1371/journal.pone.0153047)
Supplement: S1 Appendix — (PDF) [file pone.0153047.s001.pdf]

Appendix 1 McMonnies Dry Eye Questionnaire

Scores for Grading are Located Next to Questions

Age

☐Under 25 Years — Male or Female 25 Years: 0 points

☐25–45 Years — Male 25–45 Years: 1 point

☐Over 45 Years — Female 25–45 Years: 3 points

Male over 45 Years: 2 points

Gender: ☐Male ☐ Female — Female over 45: 6 points

1. Have you ever had drops prescribed or other treatment for dry eyes?

☐ Yes (6 points) ☐ No (0 points) ☐ Uncertain (0 points)

2. Do you experience any of the following (if Yes, refer to question #3):

☐Soreness ☐Scratchiness ☐Dryness ☐Grittiness ☐Burning

3. How often do your eyes have these symptoms?

☐Never (0 points) ☐Sometimes (1 point) ☐Often (4 points) ☐Constantly (8 points)

4. Do you regard your eyes as being especially sensitive to cigarette smoke, smog, air conditioning, or heating?

☐Yes (0 points) ☐No (2 points) ☐Sometimes (4 points)

5. Do your eyes easily become red and irritated when swimming in chlorinated fresh water?

☐Not applicable (0 points) ☐Yes (2 points) ☐No (0 points) ☐Sometimes (1 points)

6. Are your eyes dry and irritated the day after drinking alcohol?

☐Not applicable (0 points) ☐Yes (4 points) ☐No (0 points) ☐Sometimes (2 points)

7. Do you take:

☐Antihistamine eye drops ☐Diuretics (fluid tablets)

☐Sleeping tablets ☐Tranquilizers

☐Oral contraceptives

Antihistamines (oral or drop) and/or diuretics and/or sleeping tablets and/or tranquilizers and/or contraceptives = 2 points total

☐Medication for duodenal ulcer

☐Medication for digestive problems

☐Medication for high blood pressure

Ulcer and/or digestive and/or blood pressure medication = 1 point total

☐Other _______________

8. Do you suffer from arthritis?

☐Yes (2 points) ☐No (0 points) ☐Uncertain (0 points)

9. Do you experience dryness of the nose, mouth, throat, chest, or vagina?

☐Never (0 points) ☐Sometimes (1 points) ☐Often (2 points) ☐Constantly(4 points)

10. Do you suffer from thyroid abnormality?

☐Yes (2 points) ☐No (0 points) ☐Uncertain (0 points)

11. Are you known to sleep with your eyes partly open?

☐Yes (2 points) ☐No (0 points) ☐Uncertain (0 points)

12. Do you have eye irritation when you wake up from sleeping?

☐Yes (2 points) ☐No (0 points) ☐Uncertain (0 points)

From McMonnies CW.Key questions in a dry eye history. J Am Optom Assoc 1986; 57:513–7.Copyright©1986 by the American Optometric Association. Reprinted with permission.
