# Supplementary material for: Accuracy of McMonnies Questionnaire as a Screening Tool for Chinese Ophthalmic Outpatients
Source: PLoS One. 2016 Apr 13;11(4):e0153047. doi: 10.1371/journal.pone.0153047 (PMC4830624; doi:10.1371/journal.pone.0153047)
Supplement: S1 Text — (PDF) [file pone.0153047.s003.pdf]

Fourfold table of diagnosis results across the study population

MI>14.5	Gold standard diagnose		Total
	DED	Non-DED	
DED	14448	1250	15698
Non-DED	4411	7672	12083
Total	18859	8922	27781
