# Supplementary material for: Accuracy of McMonnies Questionnaire as a Screening Tool for Chinese Ophthalmic Outpatients
Source: PLoS One. 2016 Apr 13;11(4):e0153047. doi: 10.1371/journal.pone.0153047 (PMC4830624; doi:10.1371/journal.pone.0153047)
Supplement: S2 Text — (PDF) [file pone.0153047.s004.pdf]

Area Under the Curve

Test Result Variable(s): MI scores

Area	Std. Error[a]	Asymptotic Sig.[b]	Asymptotic 95% Confidence Interval	
			Lower Bound	Upper Bound
.865	.002	.000	.861	.869

The test result variable(s): MI scores has at least one tie between the positive actual state group and the negative actual state group. Statistics may be biased.

a. Under the nonparametric assumption

b. Null hypothesis: true area = 0.5

Coordinates of the Curve

Test Result Variable(s): MI scores

Positive if Greater Than or Equal To[a]	Sensitivity	1 - Specificity
-1.0000	1.000	1.000
.5000	.999	.997
1.5000	.998	.988
2.5000	.997	.965
3.5000	.994	.940
4.5000	.990	.900
5.5000	.982	.842
6.5000	.973	.774
7.5000	.958	.694
8.5000	.943	.604
9.5000	.922	.521
10.5000	.900	.448
11.5000	.873	.337
12.5000	.845	.272
13.5000	.808	.196
14.5000	.766	.140
15.5000	.687	.110
16.5000	.591	.081
17.5000	.491	.050
18.5000	.406	.031
19.5000	.334	.021
20.5000	.266	.013
21.5000	.212	.008
22.5000	.166	.005
23.5000	.129	.003
24.5000	.091	.002
25.5000	.065	.001
26.5000	.048	.001
27.5000	.034	.001

28.5000	.021	.001
29.5000	.015	.001
30.5000	.010	.000
31.5000	.007	.000
32.5000	.005	.000
33.5000	.003	.000
34.5000	.002	.000
35.5000	.002	.000
36.5000	.001	.000
37.5000	.001	.000
38.5000	.001	.000
39.5000	.000	.000
40.5000	.000	.000
41.5000	.000	.000
43.0000	.000	.000

The test result variable(s): MI scores has at least one tie between the positive actual state group and the negative actual state group.

a. The smallest cutoff value is the minimum observed test value minus 1, and the largest cutoff value is the maximum observed test value plus 1. All the other cutoff values are the averages of two consecutive ordered observed test values.
